# Comparative analysis of microflora and antibiotic resistance in patients with sepsis in 1999 and 2013

**DOI:** 10.1186/cc14170

**Published:** 2015-03-16

**Authors:** IV Avdoshin, LG Akinchits, ES Konstantinova, MA Shatil, ON Dobrydin, NA Bubnova

**Affiliations:** 1 Saint-Petersburg City Hospital of St. George, Saint-Petersburg, Russia

## Introduction

Changes in infection agents and their sensitivity to antibiotics are the main cause of severity of surgical infections. In spite of development and Introduction of new drugs and methods of treatment, the number of patients with sepsis increases, so the problem in diagnosing and treatment is still far from resolution.

## Methods

A comparative retrospective analysis of 52 histories of patients with sepsis, which were treated in the Department of Surgical Infections in 1999 and 2013.

## Results

The number of patients with sepsis in 2013 was raised 2.7 times, in comparison with 1999. Mortality decreased from 79% in 1999 to 55% in 2013. In most cases sepsis was accompanied with immunosuppressive disorders, such as diabetes, oncology, alcohol and drug addiction, and HIV infection. We analyzed crops of discharge from the wound and blood cultures in 52 patients with sepsis. Crops of wound were taken during the initial surgical intervention, then every 3 to 7 days, as well as the surgical interventions being repeated. Blood cultures were performed in the presence or suspected diagnosis of sepsis, in accordance with the classification Bone criteria. In comparison of spectrum of infection agents, *Staphylococcus aureus *is still leading (1999 - 36.6% of isolates, 2013 - 25%), and the percentage of MRSA was 0% in 1999 and 37.5% in 2013. The frequency of Gram-negative flora has increased: *E. coli *(8.5%/20%), *P. aeruginosa *(8.5%/12%), *Klebsiella pneumoniae *(0%/16%) and *Acinetobacter *spp. (0%/16%). Speaking about the resistance of microorganisms, there is still a high percentage of sensitivity to aminoglycoside antibiotics (79.4%/75%), glycopeptides (77.2%/71%), carbapenems (88.4%/78%) and also to the combination therapy (71.8%/62.4%), but also a reduction in sensitivity to the group of beta-lactam antibiotics (58.2%/32.5%) and fluoroquinolones (64.6%/36.4%).

## Conclusion

The number of patients with sepsis has increased; the mortality of sepsis has decreased. The frequency of *S. aureus *isolation is still high, MRSA is the same. The frequency of Gram-negative flora isolation has increased, especially *K. pneumoniae *and *Acinetobacter *spp. The resistance of microorganisms to beta-lactams and fluoroquinolones is rising but the sensitivity to aminoglycosides, glycopeptides, and carbapenems is still maintained.

